# MRI Brain Findings and Clinical Spectrum of Children With Typhus-Positive Acute Encephalitis Syndrome: Associations With Neurological Sequelae

**DOI:** 10.7759/cureus.104257

**Published:** 2026-02-25

**Authors:** Anita Mehta, Saeed Gani, Vijai Shankar, Ankur Kumar, Kaleem Ahmad

**Affiliations:** 1 Pediatrics, Baba Raghav Das Medical College, Gorakhpur, Gorakhpur, IND; 2 Pediatrics, Autonomous State Medical College, Gonda, IND; 3 Surgery, Keshav Memorial Charity Medical College, Maharajganj, IND; 4 Microbiology, Baba Raghav Das Medical College, Gorakhpur, Gorakhpur, IND; 5 Radiology, Baba Raghav Das Medical College, Gorakhpur, Gorakhpur, IND

**Keywords:** acute encephalitis syndrome (aes), magnetic resonance imaging (mri), myocarditis, orientia tsutsugamushi, pediatric intensive care unit (picu), refractory seizure, scrub typhus, sequelae

## Abstract

Background

Scrub typhus, caused by *Orientia tsutsugamushi* through chigger bites, is a significant cause of acute encephalitis syndrome (AES) in children in Southeast Asia.

Materials and methods

This cross-sectional study was conducted in the pediatric intensive care unit (PICU) of a tertiary care hospital in Gorakhpur district, Uttar Pradesh, India, over one year, from October 2021 to September 2022.

Result

Among 274 AES patients screened, 144 (52.55%) tested positive for scrub typhus via enzyme-linked immunosorbent assay (ELISA). Half of the affected patients were aged 1-5 years. MRI abnormalities were found in 29 (27.10%) patients, showing multiple patchy lesions in both cerebral hemispheres, caudate nucleus, basal ganglia, thalamus, and the midbrain. Significant associations were identified between shock (p=0.00001), myocarditis (p=0.002), refractory seizure (p=0.001), respiratory failure (p=0.001), and the development of sequelae (p=0.0003) at discharge.

Conclusion

Scrub typhus is the leading cause of AES in North India. Abnormal MRI findings have a significant association with complications and sequelae at discharge in AES patients.

## Introduction

Scrub typhus is an acute febrile illness (AFI) endemic to the "tsutsugamushi triangle," covering much of South and Southeast Asia, the Asian Pacific Rim, and northern Australia, an area inhabited by over one billion people [1]. It is caused by *Orientia tsutsugamushi *(*O. tsutsugamushi*), an obligate intracellular gram-negative bacterium formerly classified under *Rickettsia*. Multisystem involvement increases the risk of fatality, underscoring the importance of early antibiotic therapy [2].

Although the prevalence of the disease was rare, recent outbreaks across several Indian states highlight its re-emergence [3]. In eastern Uttar Pradesh, *O. tsutsugamushi* has become a leading cause of acute encephalitis syndrome (AES), emphasizing the need for prompt diagnosis and management [2,3].

AES outbreaks in Gorakhpur district in the Indian state of Uttar Pradesh have recurred for more than two decades, with high case fatality rates, particularly during the monsoon and post-monsoon seasons, mostly affecting children. Scrub typhus accounts for nearly two-thirds of AES cases and is also a major cause of AFI in this region [2,4].

Transmission occurs through the bite of trombiculid mites inhabiting moist vegetated soil. Risk factors include proximity to grasslands, vegetable fields, or ditches; mud floors; indoor vegetation; and nearby scrub areas, along with certain occupational practices. Because of its nonspecific and variable presentation, scrub typhus is difficult to diagnose clinically. Both laboratory and radiological investigations aid diagnosis, with studies reporting scrub typhus as the etiology in up to 66.7% of AES cases [2,3,5].

Susceptibility-weighted and diffusion-weighted MRI sequences can identify brain lesions such as edema, signal alterations, diffusion restriction, hemorrhage, necrosis, and enhancement. In some instances, MRI can suggest a specific AES etiology [5,6]. This study, therefore, aimed to evaluate MRI brain findings in serologically confirmed scrub typhus-related AES cases and to examine their associations with complications and sequelae at discharge.

## Materials and methods

This cross-sectional observational study was conducted over a period of one year in the pediatric intensive care unit (PICU) of Baba Raghav Das Medical College, Gorakhpur, a tertiary care hospital in eastern Uttar Pradesh, India. Patient data were collected from October 2021 to March 2022, and analysis was performed in the next six months. We screened all patients with AES aged 1-18 years who presented to our emergency department. AES was defined as an acute onset of fever of less than seven days accompanied by a change in mental status or new onset seizures (excluding febrile seizures), with cerebrospinal fluid pleocytosis (cell counts >5/mm^3^) and raised protein [7]. These AES patients were investigated for etiology, i.e., Japanese encephalitis (JE), scrub typhus, dengue, malaria, chikungunya, and *Leptospira* in the college microbiology and virology labs of the Regional Medical Research Centre (RMRC), a regional branch of the Indian Council of Medical Research (ICMR) situated in the premises of our medical college. All consecutive scrub typhus-positive patients identified by the enzyme-linked immunosorbent assay (ELISA) method were enrolled in the study after obtaining parental consent. The modified Kuppuswami scale was used to define the socioeconomic status of patients' family details [8]. These patients were managed according to our national guidelines [9].

Investigations

A total of 3 ml of blood was taken in an ethylenediaminetetraacetic acid (EDTA) vacutainer and 3 ml in a plain vial for complete blood count (CBC), C-reactive protein (CRP), serum electrolytes, liver function test (LFT), kidney function test (KFT), HIV, HBsAg, HCV, typhoid IgM, CSF studies, and serum glucose. All these tests were done within the departmental side lab. Electrocardiography was done within the department, and EEG was done in the Neurology department. X-ray chest and ultrasonography (USG) were done in the Radiology department of our college.

Once the patients were stabilized, an MRI was performed, typically 7-10 days after admission. All patients underwent T1W, T2W, FLAIR, axial diffusion-weighted imaging (DWI), and ADC sequences.

Diagnosis of scrub typhus and other etiologies of AES

We collected 3 mL of blood in clot activator test tubes and sent it immediately in ice packs to RMRC, otherwise refrigerated. The serum and CSF were tested for *O. tsutsugamushi* IgM using a commercial ELISA (Scrub Typhus Detect; InBios International Inc., Seattle, WA) kit. We considered an optical density value >0.5 to be a positive result [10]. The samples were also tested for dengue (Panbio Dengue IgM capture ELISA, Brisbane, Australia), chikungunya (IgM capture ELISA), malaria (MERISCREEN Malaria Pf/Pv Ab: Meril Diagnostics), and *Leptospira* (Panbio Leptospira IgM ELISA, Brisbane, Australia), using commercial enzyme-linked immunosorbent assays (ELISA). Ethical approval was obtained from the Institute Ethics Committee (IEC) with reference no 34/IHEC/2023.

Data analysis

IBM SPSS Statistics version 25.0 (IBM Corp., Armonk, NY) was used for data analysis. Data were expressed as frequencies and percentages. The chi-square test was used to analyze qualitative variables, and a p-value <0.05 was considered statistically significant.

## Results

The present study, conducted at a tertiary care teaching hospital from October 2021 to March 2022, investigated the clinical profile and MRI brain findings in scrub typhus-positive AES patients, and their association with complications and sequelae at discharge. Among 274 AES patients screened, 144 (52.55%) were positive for scrub typhus, 36 (12.6%) for Japanese encephalitis, and five (1.8%) each for dengue and chikungunya, while no malaria cases were detected. A total of 84 patients (30.1%) remained undifferentiated. Of the 144 scrub typhus-positive AES patients, 107 were included in the final analysis; 37 were excluded (31 due to inability to undergo MRI and six due to parental refusal).

Demographic data show that nearly half of the scrub typhus AES patients were aged 1-5 years, and the majority (n=90, 84.1%) were below 10 years of age. Only seven patients (6.5%) were older than 15 years. The male-female ratio was 1:1.4, and most patients (n=86, 80.4%) belonged to rural areas (Table 1).

**Table 1:** Demographic profile of scrub typhus-positive AES patients AES, acute encephalitis syndrome

Variables		Number (n=107)	Percentage (%)
Age	1-5	53	49.53
	5-10	37	34.58
	10-15	10	9.35
	>15	7	6.54
Gender	Male	50	46.73
	Female	57	53.27
Socioeconomic status	Upper	6	5.60
	Upper middle	15	14.01
	Lower middle	54	50.47
	Upper lower	17	15.89
	Lower	15	14.01
Residence	Rural	86	80.37
	Urban	21	19.62

Table 2 presents the clinical features and investigations of scrub typhus-positive AES patients. Headache, vomiting, and anemia were reported in 45 (42.06%), 42 (39.25%), and 46 (42.99%) patients, respectively. Seizures were noted in 83 (77.57%) of the scrub typhus-positive AES patients, with the types being focal motor in 7 (6.54%) and generalized tonic-clonic in 76 (71.03%) patients. Refractory seizures occurred in 6 (5.61%) patients. No myoclonic seizures were observed in the study population. CSF was clear in appearance in all the patients, protein was less than 100 mg/dl in 81.31% patients, sugar was normal in the majority (83.18%) of cases, and mild mononuclear pleocytosis (<100 cells/mm^3^) was seen (Table 2).

Abnormal X-ray findings were observed in 22 (20.56%) of the scrub typhus-positive AES patients. These findings included heterogeneous alveolar opacity, cardiomegaly, bilateral mild pleural effusion, bilateral pulmonary edema with a butterfly-like distribution suggestive of acute respiratory distress syndrome, bilateral diffuse reticulonodular opacities, and peribronchial thickening.

**Table 2:** Clinical features and investigations of scrub typhus-positive AES patients AES, acute encephalitis syndrome, GTCS, generalized tonic–clonic seizure; SGOT, serum glutamic oxaloacetic transaminase; SGPT, serum glutamic pyruvic transaminase; LFT, liver function test; PT/INR, prothrombin time/international normalized ratio; ABG, arterial blood gas; CPK-MB, creatine kinase-MB; CRP, C-reactive protein

Clinical feature		Frequency (n=107)	Percentage (%)
Fever		107	100
Altered sensorium		107	100
Headache		45	42.06
Vomiting		42	39.25
Anemia		46	42.99
Seizure			
Focal		7	6.54
Myoclonic		0	0
GTCS		76	71.03
Edema		20	18.69
Rashes		10	9.35
Eschar		6	5.6
Lymphadenopathy		25	23.36
Splenomegaly		18	16.82
Hepatomegaly		35	32.71
Respiratory distress		30	28.04
Abnormal LFT		48	44.86
Raised serum creatinine		11	10.28
Leukocytosis		51	47.66
Thrombocytopenia (<50,000)		18	16.82
Raised PT/INR		13	12.15
Abnormal ABG		62	57.94
Raised CPKMB		23	21.5
Raised CRP		81	75.7
Abnormal X-rays		22	20.56

Table 3 details the complications experienced by the scrub typhus-positive AES patients. The most common complication was shock, occurring in 39 (36.45%) patients, followed by hepatitis in 29 (27.10%), myocarditis in 23 (21.5%), and pneumonia in 22 (20.56%) patients. Other complications included thrombocytopenia (<100,000) in 70 (65.42 %), acute kidney injury (AKI) in 11 (10.28%), respiratory failure in 8 (7.47%), and diffuse intravascular coagulopathy in 7 (6.54%) patients (Table 3).

**Table 3:** Complications of scrub typhus-positive AES patients AES, acute encephalitis syndrome; AKI, acute kidney injury; DIC, disseminated intravascular coagulation; MODS, multiorgan dysfunction syndrome

Type of complication	Frequency (n=107)	Percentage (%)
Shock	39	36.45
Hepatitis	29	27.10
Myocarditis	23	21.5
Pneumonia	22	20.56
Thrombocytopenia (<50,000)	18	16.82
AKI	11	10.28
Respiratory failure	8	7.47
DIC	7	6.54
MODS	14	13.08

Table 4 displays the MRI brain findings in scrub typhus-positive AES patients. Out of 107 MRIs done, abnormalities were detected in 29 (27.10%) patients only, presenting as multiple variable-sized patchy lesions affecting both cerebral hemispheres as well as other parts of the brain. MRI sequence showed area of altered signal intensity (hypointensity on TW1, hyperintensity on TW2, and FLAIR) in 27 (25.23%) patients, restriction on diffusion-weighted images (DWI) in 25 (23.36%) patients, and hemorrhage (blooming) on susceptibility weighted (SWI) image in one (0.93%) patient. The areas of the brain involved were the fronto-temporo-occipital lobes in 22 (20.56%) patients (multifocal patches of altered signal intensity predominantly involving bilateral basi-frontal region, perisylvian cortex while nearly diffuse involvement of right temporal lobe with relative sparing of medial temporal lobe), caudate nucleus in 5 (4.67%) patients, other parts of basal ganglia in 5 (4.67%) patients, thalamus in 3 (2.80%) patients, and midbrain in 2 (1.87%) patients (Table 4).

**Table 4:** Multiple response analysis of MRI brain findings in scrub typhus–positive AES patients AES, acute encephalitis syndrome; MRI, magnetic resonance imaging; T1W, T1-weighted; T2W, T2-weighted; FLAIR, fluid-attenuated inversion recovery; DWI, diffusion-weighted imaging; SWI, susceptibility-weighted imaging

Brain imaging observation		Frequency (n=107)	Percentage (%)
Signal intensity on various MRI sequences	Hypointensity on T1W, hyperintensity on T2W and FLAIR	27	25.23
	Restriction on DWI	25	23.36
	Hemorrhage (blooming on SWI)	1	0.93
Site of brain involved	Fronto-temporo-parieto-occipital lobe	22	20.56
	Basal ganglia	10	9.35
	Thalamus	3	2.8
	Mid-brain	2	1.87

We sought to identify an association between MRI abnormalities and other variables. We found a statistically significant association between abnormal MRI findings and shock (p=0.00001), myocarditis (p=0.002), refractory seizure (p=0.001), respiratory failure (p=0.001), and development of sequelae (p=0.0003).

**Figure 1:**
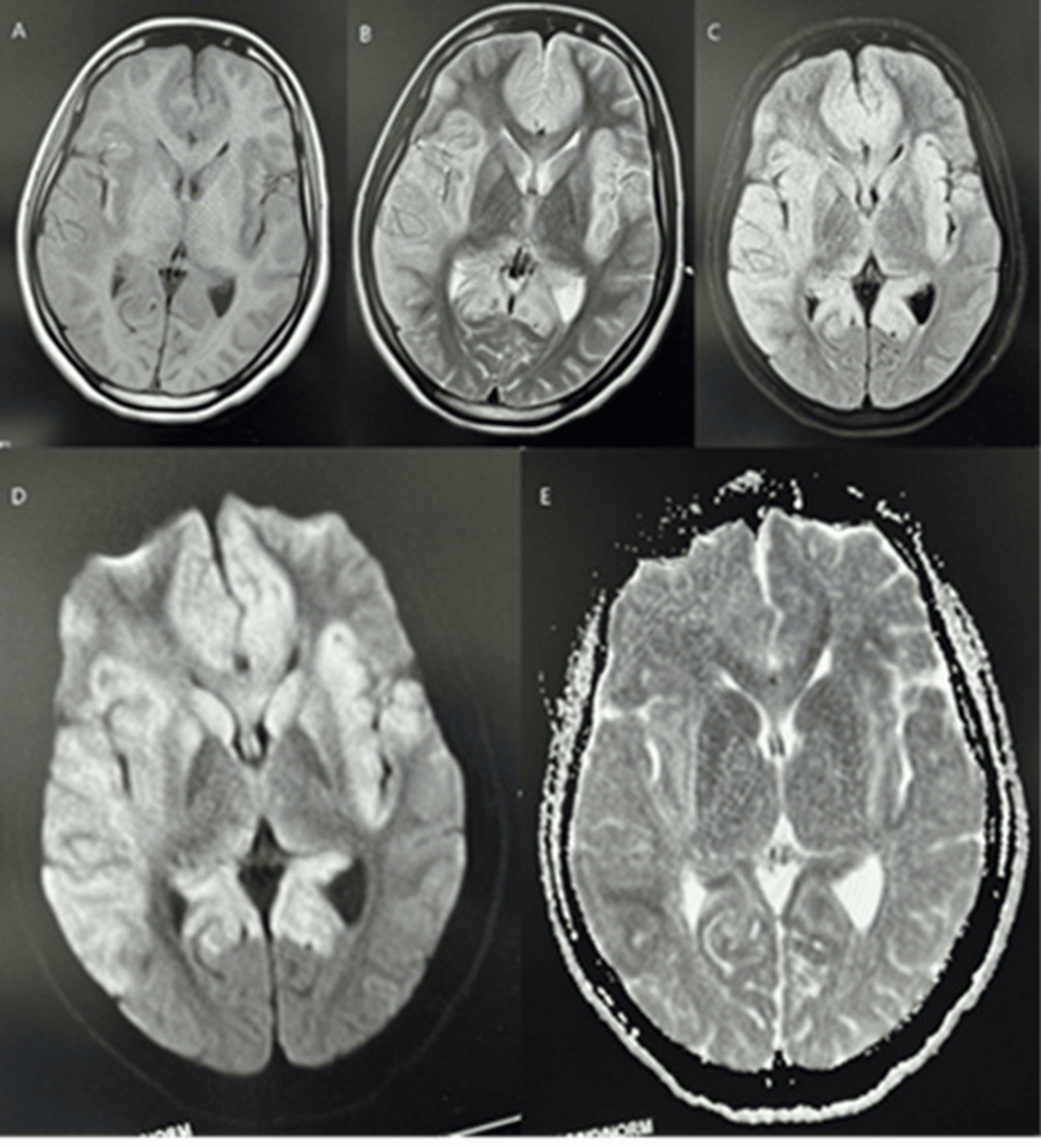
Altered signal intensity on various MRI sequences A. Axial T1-weighted (T1W) sequence shows subtle enhancement in the bilateral frontotemporal lobes, involving the basifrontal regions, perisylvian cortices, and the right temporal lobe, with relative sparing of the medial temporal lobes. B. Axial T2-weighted (T2W) sequence demonstrates multifocal hyperintense patches in the bilateral frontotemporal lobes, including the basifrontal regions, perisylvian cortices, and nearly diffuse involvement of the right temporal lobe, with relative sparing of the medial temporal lobe. Hyperintensities are also noted in deep gray matter nuclei, including the bilateral caudate and lentiform nuclei. C. Axial FLAIR sequence reveals hyperintensity in the bilateral frontotemporal lobes, involving the basifrontal regions, perisylvian cortices, and near-diffuse involvement of the right temporal lobe, with relative sparing of the medial temporal lobe. Similar signal changes are seen in the bilateral caudate and lentiform nuclei. D. Axial diffusion-weighted imaging (DWI) sequence shows hyperintensity in the bilateral frontotemporal lobes, predominantly in the basifrontal regions and perisylvian cortices, with near-diffuse involvement of the right temporal lobe and relative sparing of the medial temporal lobe. Hyperintense signals are also observed in the bilateral caudate and lentiform nuclei. E. Apparent diffusion coefficient (ADC) sequence shows corresponding hypointensity in the same regions—bilateral frontotemporal lobes (including basifrontal regions and perisylvian cortices), with near-diffuse involvement of the right temporal lobe and relative sparing of the medial temporal lobe—suggestive of restricted diffusion. Deep gray matter nuclei, such as the bilateral caudate and lentiform nuclei, are similarly involved.

Figure 1a-1b shows subtle enhancement on TW1 in bilateral fronto-temporal lobes corresponding to multifocal patches of hyperintensity on TW2 in bilateral fronto-temporal lobes involving the bilateral basifrontal region, perisylvian cortex, and near diffuse involvement of the right temporal lobe with relative sparing of the medial temporal lobe, deep gray matter nuclei such as bilateral caudate and lentiform nuclei. Figures 1c-1d show hyperintensity in similar locations of the brain on FLAIR and axial diffusion weighted (DWI) sequence, respectively. Figure 1e shows hypointensity suggestive of restricted diffusion in similar locations of the brain.

Table 5 illustrates the association between sequelae at discharge and other variables. We found no association between sequelae at discharge and male gender, refractory seizure, and respiratory failure. Our analysis indicates that shock (p=0.0007) and myocarditis (p=0.009) are statistically significantly associated with the development of sequelae in AES patients (Table 5).

**Table 5:** Association between sequelae at discharge and variables

Variables	Sequelae at discharge n=(%)		Chi-squared test	
	Present, n= (%)	Absent, n= (%)	χ2	P-value
Male gender	2(4.0)	48(96.0)	2.37	0.12
Shock	8(20.5)	31(79.5)	11.29	0.0007
Myocarditis	5(21.7)	18(78.3)	6.75	0.009
Refractory seizure	1(16.66%)	5(83.33)	0.56	0.453
Respiratory failure	8(8.1)	91(91.9)	0.19	0.664

## Discussion

Scrub typhus is an emerging disease of growing clinical significance in India and has been identified as a major cause of AES, with a range of 60-70% reported in previous studies. Central nervous system involvement in pediatric cases is well-documented, ranging from aseptic meningitis to meningoencephalitis [11,12].

In the current analysis, the positivity rate declined to 52.55%, compared to the 63% reported by Mittal et al. This reduction may reflect improved awareness, early diagnosis via a multidimensional approach, and prompt treatment by clinicians [11].

Most cases occurred during the post-monsoon period (September to November), which coincides with peak vegetation growth and increased mite populations. Similar seasonal trends have been reported by other researchers [12-14].

The most affected age group was children under five years, consistent with previous studies. In the current study, 53.3% of patients were female and 46.7% male, yielding a male-female ratio of 1:1.4, in concordance with findings by Ghimire et al. and Panda et al. [14,15].

A majority of patients (n=86, 80%) were from rural areas, suggesting frequent exposure to *O. tsutsugamushi* in the rural regions of Gorakhpur and surrounding areas. This observation is consistent with findings by Mittal et al. and Bithu et al. [11,16].

Hepatomegaly was seen in 32.7% of patients, closely matching the 34.2% reported by Behera et al. [17]. Rashes were present in 9.3% of cases, aligning with previous reports ranging from 5% to 27% [16-18]. Lymphadenopathy was noted in 23.4% of patients, similar to the 25.4% observed by Behera et al. [17]. Additionally, 28% experienced respiratory distress, comparable to Ganesh et al. reported 42% in their study in South India [18].

Fever was observed in all patients (100%), establishing it as a universal symptom. Other common symptoms included headache (42.06%), vomiting (39.25%), and anemia (42.99%). Seizures were reported in 83 patients (77.57%), consistent with findings by Borkakoty et al. and Kispotta et al., who also noted a seizure rate of 77.5% [19,20].

In the present study, thrombocytopenia was present in 65.42% patients, which is comparable to the previous study by Basu et al. [21]. Other common findings were elevated serum transaminase levels and leukocytosis, consistent with those reported by Varghese et al. and Basu et al. [3,21].

In the present study, shock was observed in 36.4% of patients, compared to 25.8% in a study by Bhat et al. [13]. Other major complications included hepatitis (27.10%), myocarditis (21.5%), and pneumonia (20.56%). Previous studies by Wangrangsimakul et al. and Pathak et al. showed similar findings [22,23].

In the current study, brain MRI was performed on 107 scrub typhus-positive AES patients, of whom 29 (27.10%) showed abnormal findings. Altered signal intensity, hypointense on T1-weighted, hyperintense on T2-weighted, and FLAIR sequences was observed in 25.23% of these cases. Diffusion restriction on DWI was seen in 23.36%, while hemorrhage (blooming on SWI) was present in 0.93%. Neyaz et al. and Kim et al. reported similar imaging features in their case reports [24,25]. The brain regions most commonly involved were the fronto-parieto-temporo-occipital lobes (20.56%), caudate nucleus and other basal ganglia structures (4.67% each), thalamus (2.80%), and midbrain (1.87%). Kim et al. reported involvement of the pontomedullary region, bilateral cerebellar peduncles, and cervical spinal cord, while Chua et al. described ring-enhancing lesions in the corpus callosum and hyperintensities in the periventricular and deep white matter on T2 and FLAIR sequences [24,26]. Kar et al., in a study of 20 adult patients, observed similar hyperintensities involving the putamen and thalamus [27].

Limitations

Firstly, the study was conducted at a single tertiary care hospital in eastern Uttar Pradesh, which may limit the generalizability of the findings to other regions with different epidemiological patterns of scrub typhus. Secondly, not all patients underwent brain MRI due to logistical and clinical constraints, potentially leading to an underestimation of the true prevalence of neurological abnormalities. Future multicentric studies with larger sample sizes, molecular diagnostic confirmation, comprehensive neuroimaging, and long-term follow-up are recommended for a better understanding of the full spectrum of scrub typhus-associated AES and its neurological outcomes.

## Conclusions

Scrub typhus has emerged as the leading cause of AES in endemic regions such as the Gorakhpur division of Eastern Uttar Pradesh, particularly among children. Early diagnosis of AES is essential to detect neurological and other sequelae, allowing for accurate clinical and diagnostic correlation. Through widespread use of diagnostic tests and neuroimaging, clinicians have observed a variety of clinical presentations and characteristic MRI findings in affected pediatric patients. Raising awareness among clinicians in endemic areas, along with the development of rapid diagnostic methods, is crucial for improving patient outcomes and reducing mortality linked to this serious disease.
